# Supplementary material for: Examining the availability/findability of stimuli employed in social media and body image research
Source: PLoS One. 2025 May 22;20(5):e0324514. doi: 10.1371/journal.pone.0324514 (PMC12097549; doi:10.1371/journal.pone.0324514)
Supplement: Attachment — Submitted filename: rebuttal_letter_resubmission01_v3.docx [file pone.0324514.s001.docx]

**Rebuttal Letter**

Thank you for the helpful comments. Please find our response to the reviewers’ comments below.

**Reviewer #1: Peer Review**

**Comment 1. (page 3) I would suggest moving the explanation of ‘open research’ practices directly after the sentences where you introduce the concept (phrase ends with “in a set of ‘open research’ practices’**

Response 1 = Thanks for this comment. We have revised the manuscript in line with this suggestion, so that our argument/description is clearer. The revised paragraph is presented below (we have moved the ‘struck-through’ text so that it is now the second, rather than the final, sentence in this paragraph): “Since 2012, and the start of the so-called ‘replication crisis’ (Lillenfield & Strother, 2020), Psychology researchers (as well as researchers in other disciplines, e.g., biology; see Allen & Mehler, 2019) have increasingly engaged in a set of ‘open research’ practices. ‘Open research’ practices include the pre-registration of predictions and analysis plans (e.g., Nosek et al., 2019), the use of open-source software (e.g., Kathawhalla et al., 2021), and the open sharing of data (e.g., Rouder, 2016). By adopting these practices, individuals aim to increase the transparency and rigor of their research. As a result, the likelihood that they will generate robust, replicable findings should be increased. ~~‘Open research’ practices include the pre-registration of predictions and analysis plans (e.g., Nosek et al., 2019), the use of open-source software (e.g., Kathawhalla et al., 2021), and the open sharing of data (e.g., Rouder, 2016).~~”

**Comment 2. Please describe how the articles were selected. Did you choose them randomly or did you apply a certain set of criteria?**

Response 2 = Apologies that this wasn’t clearer in the original manuscript. Our approach involved ‘re-using’ the sample of articles synthesized in de Valle et al.’s (2021) meta-analysis on the topic of how exposure to images from social media affects body image (which we think is a sensible thing to do, as it reduces research waste). We reviewed all 36 articles synthesized in that meta-analysis. We have made a minor edit to the ‘Sample of Articles’ sub-section to make this clearer.

**Comment 3. (page 5) When you write about ‘female participant’ do you mean cisgender females? Do you refer only to the biological sex? If the studies themselves do not differentiate this would be important to note.**

Response 3 = Apologies, yes. In the places where we have used the term ‘female participants’ we have relied on the reporting in the original papers/de Valle et al.’s meta-analysis. We have, therefore, revised our manuscript so that in both places (page 5 and page 6) we have changed from using the term “female participants” to “participants who self-reported their gender as female” (as this is the terminology used in the De Valle et al. meta-analysis).

**Comment 4. Please also explain how you extracted the general information of the articles. Did you use an extraction shed? If so, I would suggest providing the table in the appendix.**

Response 4 = Apologies, but we are not familiar with the term ‘extraction shed’. The document available for download at https://osf.io/96rwx is a copy of the document we used to code the publications. We have added information directed towards readers in a sheet titled ‘Notes’, with ‘Sheet 1’ containing the document we used to code the publications.

**Comment 5. (page 5) Please explain how you generated the code system. Was it generated based on literature (deductively) or based on the included articles (inductively)? Or was it based on your perceptions? If the latter, please describe your potential influence (bias) on the creation of the categories.**

Response 5 = The coding system was shaped by our impressions of common practices in the literature, based on what two of us (DS and AA) had noticed when planning a study that examined the effect of exposure to images from social media on women (and we perceived that the sharing of stimuli was very uncommon). We have added text to the Method section (see page 5) to provide clarity on this: “~~After refining a draft~~ We drafted a coding system based on our past experiences of reading papers that investigated the effect of exposure to images from social media on participants’ body image.” In addition, we have noted that this may be considered to be a limitation in the Discussion section: “Third, we developed our coding system based on our past experience of reading publications in this field, rather than using coding systems developed by other researchers. This may have introduced some bias in, for example, the codes generated.”

**Comment 6. On how many studies was the code system piloted? And how where the studies chosen?**

Response 6 = The initial coding system was piloted on all of the eligible studies. We have added text to the Method section (see page 5) to provide clarity on this: “One author (JV) piloted the coding system on all 36 studies. After this pilot, we refined the coding system (e.g., making some language more precise, revising some examples), and then all studies were reviewed and coded by two authors (DS and MC) using this revised coding scheme, to establish inter-rater reliability.”

**Comment 7. I would suggest reporting the inter-rater-reliability directly in the methods session.**

Response 7 = We have moved the reporting of inter-rater reliability into the Method, in line with this suggestion.

**Comment 8. Further, please provide an overview of the codebook you used as supplementary material, including the codes and maybe one or two coded text passages from the text. This could be helpful for an overview.**

Response 8 = Apologies – we did make the coding scheme (we assume that ‘codebook’ refers to the coding scheme employed. But apologies if we are wrong about that) available (via the OSF) at the time of submission, but the link to the coding scheme was not very obvious in the manuscript. We have now included a link to the coding scheme nearer to the start of the Method section (see page 6), whereas previously the link was provided close to the end of the Method section.

**Comment 9. (page 7, line 2) Was the question mark behind “results” intended?**

Response 9 = Apologies for this typo. The ‘?’ has been replaced by a ‘.’ in the re-submitted manuscript.

**Comment 10. Please explain why you did not the reliability for all codes?**

Response 10 = We have included measures of inter-rater reliability for all codes. We have added the following text to the Method section: In addition, we examined level of agreement code-by-code (i.e., Did DS and MC both score ‘yes’ or both score ‘no’ for Code 1? Did DS and MC both score ‘yes’ or both score ‘no’ for Code 2? And so on). For Code 1, there was perfect agreement (both raters scored ‘yes’ for all studies). For Code 2, we found ‘substantial’ levels of agreement (same scores given for 39 of 41 studies; kappa = 0.88). For Code 3 (same scores given for 38 of 41 studies; kappa = 0.78) and Code 4 (same scores given for 40 of 41 studies; kappa = 0.79), we found ‘moderate’ levels of agreement.

**Comment 11. Please provide the references of the studies you are referring to (e.g. “We rated two studies (XZY et al.; ZTU et al.)**

Response 11 = We have now provided these references, in line with the suggestion (see page 09: “We rated two studies (reported across two publications; Brichacek et al., 2018; Taniguchi et al., 2012) as sharing images/providing links to images in a way that made their task stimuli fully reproducible…”).

**Reviewer #2: Peer Review**

**The manuscript clearly outlines the study's objective, which is to examine the availability of images used as stimuli in research on the impact of social media images on body image. This is an important topic, particularly due to the increased use of social media since the pandemic and its association with mental health issues. Additionally, it emphasizes the significance of the Open Science Framework for result reproducibility and contributing to scientific integrity.**

**The manuscript provides a clear and detailed methodology for assessing the availability of images used in research on the impact of social media on body image. It includes details on article selection and acknowledges the sample size limitation. The dependency on other researchers' search strategies is mentioned as a limitation, and discussing potential associated biases is suggested. The methodology addresses result reproducibility and generalization. It is recommended to provide details on the application of statistical methods and discuss the temporal perspective regarding potential changes in practices. Overall, the methodology is robust, but improvements are suggested to enhance understanding and study validity.**

**The results indicate a significant lack of complete availability of images used in the reviewed studies, emphasizing that most articles provide general and imprecise descriptions rather than sharing the images. The manuscript contextualizes the findings within previous research in psychology, highlighting the low proportion of shared research materials across different disciplines. It includes appropriate supplementary material.**

**The implications of not sharing stimulus images are explored, including the difficulty of reproducing studies, reduced replicability, and potential resource wastage in developing similar tasks. Various factors contributing to the lack of sharing, such as a lack of incentives, complexity, and potential concerns about privacy and copyright, are suggested. Practical suggestions to address privacy and copyright concerns, such as consulting copyright librarians and using URLs with detailed descriptions, are mentioned in the manuscript.**

**The need for future research to assess whether the practice of sharing images improves over time, especially with specific interventions like journal policies and recognition systems, is highlighted. The language and style of the manuscript appear appropriate and clear.**

**Comment 12 = Note: The document mentions that attached data is available on the Open Science Framework (see https://osf.io/egamu and https://osf.io/yce9z). However, it was not possible to verify the specific content of the mentioned attachments in the document.**

Response 12 = Apologies, but we do think that these links are working. The nature of the PowerPoint files stored at those links means that the OSF doesn’t render a preview when you ‘land’ on that page. However, as best we can tell, the files stored at those pages are available for download. Please let us know if this does not seem to be the case for you.
